# Diaryl Urea Derivative Molecule Inhibits Cariogenic *Streptococcus mutans* by Affecting Exopolysaccharide Synthesis, Stress Response, and Nitrogen Metabolism

**DOI:** 10.3389/fcimb.2022.904488

**Published:** 2022-05-10

**Authors:** Ying Liao, Mengyun Zhang, Xingnan Lin, Fuhua Yan

**Affiliations:** ^1^ Department of Pediatric Dentistry, Nanjing Stomatological Hospital, Medical School of Nanjing University, Nanjing, China; ^2^ Nanjing Stomatological Hospital, Medical School of Nanjing University, Nanjing, China; ^3^ School/Hospital of Stomatology, Zhejiang Chinese Medical University, Hangzhou, China; ^4^ Department of Periodontology, Nanjing Stomatological Hospital, Medical School of Nanjing University, Nanjing, China

**Keywords:** dental caries, *Streptococcus mutans*, transcriptomic study, small molecules, stress response, nitrogen metabolism

## Abstract

Different small molecules have been developed to target cariogenic bacteria *Streptococcus mutans*. Based on target-based designing and *in silico* screening, a novel diaryl urea derivative, 1,3-bis[3,5-bis(trifluoromethyl)phenyl]urea (BPU), has previously been found effective in inhibiting the growth of *S. mutans*. However, the exact mechanism remains unclear. This current study aimed to explore the antimicrobial and antibiofilm effects of BPU on *S. mutans* and locate key enzymes and biological processes affected by the molecule *via in silico* molecular docking analysis and transcriptomic profile. Our *in vitro* results confirmed that BPU was capable of inhibiting planktonic growth as well as biofilm formation of *S. mutans*. The virtual binding analysis predicted that the molecule had strong binding potentials with vital enzymes (3AIC and 2ZID) involved in extracellular exopolysaccharide (EPS) synthesis. The predicted inhibitive binding was further confirmed by *in vitro* quantification of EPS, which found a decreased amount of EPS in the biofilms. The transcriptomic profile also found differential expression of genes involved in EPS synthesis. Moreover, the transcriptomic profile implied alterations in stress response and nitrogen metabolism in *S. mutans* treated with BPU. Examination of differentially expressed genes involved in these biological processes revealed that altered gene expression could contribute to impaired growth, biofilm formation, and competitiveness of *S. mutans*. In conclusion, the novel diaryl urea derivative BPU can inhibit the virulence of *S. mutans* by affecting different biological processes and serves as a potent anti-caries agent.

## Introduction

The prevention and treatment of dental caries have long been associated with the interference of cariogenic bacteria in the oral cavity. Traditional anti-caries agents, including fluoride and antibiotics, have been reported to contribute to local and systematic toxicity, as well as drug resistance (Liao et al., 2017; Qiu et al., 2020). Novel anti-caries chemicals are required to inhibit the growth and metabolism of cariogenic microorganisms while exerting limited side effects.

Various small molecules have been developed to target cariogenic microorganisms including the notorious species, *Streptococcus mutans* (Cui et al., 2019; Yang et al., 2021). These small molecules usually have unique structures to target specific metabolic pathways and vital enzymes in bacterial cells (Zhang et al., 2015). Different approaches, including drug repositioning, library screening, natural products screening, and target-based designing, have been applied to identify small molecules that have anti-caries potentials (Yang et al., 2021). Among them, target-based designing and *in silico* screening from the small-molecule library have been of special interest, as they provide hits with good specificity and high throughput (Younson and Kelly, 2004; Nijampatnam et al., 2016; Nijampatnam et al., 2021).

**Figure 1:**
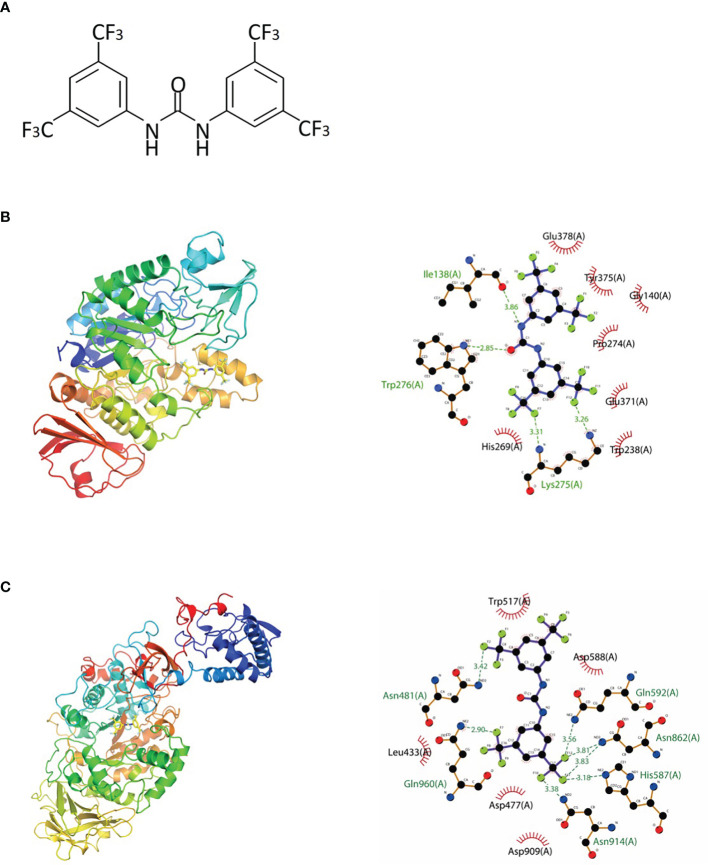
Chemical structure and molecular docking analysis. **(A)** Chemical structure of 1,3-bis[3,5-bis(trifluoromethyl)phenyl]urea (BPU) molecule. **(B, C)** Predicted binding mode (left, 3D; right, 2D) of BPU molecule with 2ZID **(B)** and 3AIC **(C)**. Hydrogen bond is shown with green dashed lines (right). Hydrophobic interaction is shown with red “eyelashes”.

Based on the abovementioned methods, a previous study identified a series of small molecules that have the potential to enhance fluoride toxicity in bacterial cells (Nelson et al., 2015). One of the small molecules, 1,3-bis[3,5-bis(trifluoromethyl)phenyl]urea (BPU; **Figure 1A**), was found to be especially effective in inhibiting the growth of *Escherichia coli* and *S. mutans* when used in combination with fluoride (Nelson et al., 2015). The enhanced antimicrobial ability was suggested to be associated with the trifluoromethyl substituents on the aryl rings, which facilitate fluoride/chloride uptake and/or retention (Busschaert et al., 2012; Nelson et al., 2015). Further investigation of the molecule structure reveals that the trifluoromethyl substituents are not the only structure related to antimicrobial effects. The diaryl rings in the small molecule contribute as hydrogen-bond donors and have been proved to be able to bind and inhibit specific bacterial proteins including DNA gyrase B and penicillin-binding protein 1a (Limban et al., 2020; Sroor et al., 2021). Moreover, similar urea derivatives have been used to target pathogenic bacteria such as *Staphylococcus aureus*, *Pseudomonas aeruginosa*, and *Enterococcus faecalis* (Wu, 1965; Gunduz et al., 2020). The preliminary structure investigation indicated that BPU has the potential to exert an inhibitive effect against *S. mutans* alone and act as a novel anti-caries agent. However, no *in vitro* result has been reported to support the antimicrobial effect of BPU alone on *S. mutans*. Also, the exact inhibition site and affected biological processes remain unknown.

In this study, we investigated the antimicrobial and antibiofilm effects of the novel diaryl urea derivative, namely, BPU, against *S. mutans* UA159. *In silico* molecular docking analysis was applied to predict the binding potential and binding site of the small molecule with different proteins in *S. mutans* cells. Transcriptomic analysis was performed to validate the predictions and locate major biological processes that were affected by the small molecule.

## Materials and Methods

### *In Silico* Molecular Docking

The three-dimensional structure of BPU was downloaded from PubChem (Cas no. 3824-74-6). The structure was geometrically optimized and energy minimized using ChemBio3D Ultra 14.0 software. A total of 20 proteins originating from *S. mutans* were selected for *in silico* molecular docking (**Table 1**). These proteins were selected because they have been reported to be associated with the virulence of *S. mutans* and their crystal structures were available online. Crystal structures of targeted proteins were obtained from Protein Data Bank (PDB) with accession codes. The protein structures were processed step by step including the removal of original ligands and water molecules, the addition of polar hydrogen atoms, and charge calculation and distribution using PyMOL v2.3.0 and AutoDocktools v1.5.6. Docking analysis was performed using AotoDock Vina v1.1.2 with default parameters. Results with the strongest protein–ligand interactions were visualized and analyzed with PyMOL v2.3.0 and LigPlot v2.2.4.

**Table 1:** Proteins selected for molecular docking study.

PDB accession	Annotation
5UQZ	Glucan binding protein C
3BJV	Putative phosphotransferase system enzyme IIA (PtxA)
6CAM	Glucan binding protein C (sucrose-dependent)
3CZC	Putative phosphotransferase system enzyme IIB (PtxB)
3QE5	C-germinal region of Antigen I/II
3AIE	Glucansucrase
3OPU	C-terminal domain of surface protein (SpaP)
2HVV	dCMP deaminase
3AIC	Glucosyltransferase (GtfC)
4TSH	Surface protein adhesin P1
3EXT	3-Keto-l-gulonate 6-phosphate decarboxylase
2ZID	Dextran glucosidase (DexB)
2RI0	Glucosamine 6-phosphate deaminase (NagB)
3L8R	Putative phosphotransferase system, cellobiose-specific IIA (PtcA)
2NQ5	Methyltransferase
3OIX	Putative dihydroorotate dehydrogenase
5ZA3	C-terminal domain of response regulator (VicR)
3PN8	Putative 6-phospho-beta-glucosidase
6TZL	Surface protein adhesin (SspB)
3K8U	Peptidase domain of putative ABC transporter, ATP-binding protein (ComA)
3M7V	Phosphopentomutase (DeoB)

### Experimental Chemicals, Bacterial Strain, and Growth Conditions

Different amount of BPU (Sigma-Aldrich, CA, USA) was dissolved in acetonitrile (Aladdin, Nanjing, China) for *in vitro* studies. *S. mutans* UA159 was obtained from the State Key Laboratory of Oral Disease (Sichuan University, Chengdu, China). Bacteria were routinely grown in brain heart infusion (BHI) broth (BD, NJ, USA) or on BHI agar plates at 37°C anaerobically (10% H_2_, 5% CO_2_, and 85% N_2_). For biofilm formation, 1% sucrose (Sigma-Aldrich, CA, USA) was added to the BHI broth.

### Planktonic Growth Assay

The antimicrobial effect of BPU on planktonic *S. mutans* UA159 was tested using a growth curve. *S. mutans* UA159 was incubated in BHI broth until the early log phase (OD_600_ = 0.2). Bacterial cells were pelleted and resuspended with fresh BHI broth containing 1, 2, 5, 10, 20, and 50 µg/ml of BPU. For blank control, fresh BHI broth containing the same volume of acetonitrile was used to resuspend bacterial cells. Resuspensions measuring 200 µl were then added into wells of a sterile 96-well microplate. The microplate was sealed with a transparent sealer and incubated at 37°C for 12 h. Growth was monitored by recording the optical density at 600 nm (OD_600_) every 30 min with Spectra MaxM3 (Molecular Devices, CA, USA).

### Crystal Violet Assay

The biomass of *S. mutans* biofilms with or without BPU treatment was quantified with a crystal violet staining assay. *S. mutans* UA159 was incubated until mid-log phase (OD_600_ = 0.5), pelleted, and resuspended with fresh BHI broth supplemented with 1% (w/w) sucrose (BHIS). Resuspensions were transferred to a sterile 96-well microplate, and BPU was added to reach different final concentrations (1, 2, 5, 10, 20, and 50 µg/ml). The same volume of acetonitrile was added to the control group. After anaerobic incubation at 37°C for 24 h, all wells were washed with sterile phosphate-buffered saline (PBS) to remove loose cells. Biofilms attached to the bottom of the wells were fixed with 4% paraformaldehyde (Sigma-Aldrich, CA, USA) for 15 min followed by staining with 0.01% crystal violet solution (Adamas, Shanghai, China) for 8 min. Solutions were then removed, and biofilms were again washed with PBS twice. Acetic acid (33% v/v, Sigma-Aldrich, CA, USA) was added to each well to destain for 10 min. The destaining solution was then transferred to a new 96-well plate, and OD_575_ was recorded with Spectra MaxM3.

### Biofilm Formation on Saliva-Coated Hydroxyapatite Discs

Biofilms of *S. mutans* UA159 were formed on saliva-coated hydroxyapatite (sHA) discs using a previously described method with a few modifications (Wang et al., 2020). Briefly, HA discs (8 mm in diameter and 2 mm in thickness; Baiameng Bioactive Materials, Chengdu, China) were sonicated in deionized water for 10 min and sterilized at 121°C for 15 min before use. Clarified saliva was prepared by centrifuging whole unstimulated saliva of a healthy human donor at 4,000 rpm for 20 min at 4°C, followed by filtration through 0.22-µm polyethersulfone membrane (Koo et al., 2010). The sterile HA discs were placed into 24-well plates. Each well contained clarified saliva mixed with the same volume of desorption buffer (containing 500 mM of KCl, 10 mM of CaCl_2_, 1 mM of MgCl_2_, 6.2 mM of K_2_HPO_4_, and 14 mM of KH_2_PO_4_). After incubation in clarified saliva and desorption buffer at 37°C for 30 min, the sHA discs were ready for use.

*S. mutans* UA159 was incubated in BHI broth until the mid-log phase (OD_600_ = 0.5). Cells were then pelleted and resuspended in BHIS. Resuspended bacterial cells were transferred to sterile 24-well plates with different concentrations of BPU or the same volume of acetonitrile. The abovementioned sHA discs were placed in each well. Plates were incubated anaerobically at 37°C for 24 h. Biofilms formed on the surface of sHA discs were used for further examinations.

### Scanning Electron Microscopy

Twenty-four-hour biofilms attached to the surface of sHA discs were harvested and washed twice with cysteine peptone water (CPW; containing yeast extract, peptone, sodium chloride, and cysteine HCl, pH 7.2). Biofilms were fixed with 2.5% glutaraldehyde (Adamas, Shanghai, China) at 4°C overnight, followed by serial dehydration with ethanol (30%, 40%, 50%, 60%, 70%, 80%, 90%, and 100%). Samples were dried and coated with gold before observation with SEM (Quanta 400 FEG, FEI, Hillsboro, OR, USA).

### pH Drop Examination

Acid production of *S. mutans* biofilms was examined with a pH drop of the supernatant. Culture media of the abovementioned biofilms measuring 2 ml formed on sHA discs were taken at 0, 4, 8, 12, and 24 h and centrifuged at 4°C at 4,000 rpm for 10 min. The pH of the supernatant was measured with an electronic pH meter (FiveEasy Plus, FE28-standard, Mettler-Toledo, Schwerzenbach, Switzerland).

### Confocal Laser Scanning Microscopy Analysis

A commercial LIVE/DEAD BacLight Viability Kit (Life Technologies, NY, USA) was used to stain viable and dead cells in 24-h biofilms. *S. mutans* biofilms formed on sHA discs were harvested after 24-h incubation. The staining of bacterial cells was processed according to the manufacturer’s instructions. Viable cells and dead cells were respectively stained with SYTO 9 (excitation, 480 nm; emission, 500 nm) and propidium iodide (PI; excitation, 490 nm; emission, 635 nm). Samples were observed with confocal laser scanning microscopy (CLSM; NikonA1; Nikon Inc., Tokyo, Japan). Images were taken at an interval of 10 µm. Integrated fluorescence density was quantified with ImageJ software (v1.48, National Institutes of Health, USA).

### Water-Insoluble Exopolysaccharide Determination

Production of water-insoluble exopolysaccharide (EPS) by *S. mutans* biofilms was examined qualitatively by fluorescence staining and quantitatively by the anthrone–sulfuric method (Tang et al., 2019). For fluorescence staining, Alexa Fluor 647 (Life Technologies, NY, USA) was used to label EPS, and SYTO 9 was used to label bacterial cells. Alexa Fluor 647 was added to the BHIS medium at the beginning of 24-h biofilm formation. Biofilms were stained with SYTO 9 at the end of the 24-h biofilm formation experiment. Samples were then observed using CLSM with the same procedure as live/dead staining.

To quantify EPS synthesis with the anthrone–sulfuric method, 24-h biofilms were harvested and scraped from sHA discs and washed with PBS buffer. The planktonic cells and suspension were removed by centrifuging the mixture at 4,000 rpm for 10 min at 4°C. The precipitate was mixed with 0.4 mol/L of NaOH and incubated for 2 h at 37°C. The mixture was centrifuged again, and the suspension was collected and transferred to a new EP tube. The suspension was mixed with three volumes of the anthrone–sulfuric acid reagent (Macklin, Shanghai, China) and heated on a heat block at 95°C for 5 min until the reaction was complete. The solution was cooled to room temperature before being transferred to a new 96-well plate. Absorbance at 625 nm was recorded, and the amount of polysaccharide was calculated according to the standard curve.

### RNA Sequencing and Data Analysis

For transcriptomic analysis, *S. mutans* UA159 was grown in BHI until the log phase (OD_600_ = 0.5). Cells were harvested and resuspended with fresh BHI. Cells were challenged with either a final concentration of 2 µg/ml of BPU or the same volume of acetonitrile for 1 h. This concentration was chosen mainly based on results from crystal violet assay to make sure that there was enough BPU to induce significant alterations in gene expression, while potential toxicity was kept as low as possible. Samples were then harvested with centrifugation and kept at −80°C until used. Total RNA was extracted using TRIzol reagent (Invitrogen, OR, USA). The concentration and quality of RNA were determined with Nanodrop 2000 spectrophotometer (Thermo Scientific, MA, USA). rRNA was removed from total RNA using Zymo-Seq RiboFree Total RNA Library Kit. cDNA library was constructed by Shanghai Personal Biotechnology Co. Ltd (Shanghai, China). RNA sequencing was performed on Novaseq 6000 platform (Illumina, San Diego, CA, USA).

Data were filtered and controlled for quality before analysis. Filtered reads were mapped to the *S. mutans* UA159 reference genome (RefSeq NC_004350.2) using Bowtie 2 (version 2.2.6). Differentially expressed genes (DEGs) were recognized as transcripts with |log_2_FoldCHange| > 1 and *p* < 0.05. Differential expression analysis was performed using DESeq (version 1.30.0). All DEGs were annotated by searching the Gene Ontology (GO) databases. All genes were mapped to terms in the GO database, and numbers of differentially enriched genes in each term were calculated. GO enrichment analysis was performed using the topGO R package on the differential genes. GO terms with significantly enriched differential genes (*p* < 0.05 according to the hypergeometric distribution method) were identified to determine the main biological functions of DEGs. Three individual samples were sequenced for each group.

### Statistical Analyses

Data were analyzed using Prism (version 9.0.0). All experiments were performed at least in triplicate. Student’s *t*-test or one-way ANOVA was employed to compare data from two or more groups. A *p*-value of <0.05 was considered statistically significant.

## Results

### *In Silico* Screening for Potential Binding Site

The binding potentials of BPU with 20 selected proteins originating from *S. mutans* are shown in **Table 2**. A higher absolute value indicates a stronger binding ability. Proteins with the highest scores were 3M7V (phosphopentomutase), 2ZID (dextran glucosidase), and 3AIC (glucosyltransferase). These three proteins were further visualized to predict the binding mode. Hydrogen bond and hydrophobic interaction acted as the main interaction forces. We further examined the potential binding sites occupied by the BPU molecule. While there was not enough information on 3M7V, we did find interesting results for 2ZID and 3AIC (**Figure 1**). The molecule can bind several vital amino acid residues of 2ZID, which were involved in the active binding pocket (Lys 275, His269, and Pro274), recognition of substrate (Glu371), and high level of enzyme activity toward long-chain substrates (Trp238) (Hondoh et al., 2008). Also, the molecule may competitively bind several conserved amino acid residues of 3AIC (His587, Asp588, Asp477, Asn481, and Asp909), which were responsible for the recognition of glucosyl moiety of the primary sucrose (Ito et al., 2011). One of the amino acid residues (Gln592) bound by the molecule was found to be involved in the catalytic domains of 3AIC (Ito et al., 2011).

**Table 2:** Binding potential with selected proteins (kcal/mol).

PDB accession	Binding potential	PDB accession	Binding potential	PDB accession	Binding potential
3M7V	−10.3	2HVV	−8.7	3QE5	−7.6
2ZID	−9.6	2NQ5	−8.7	3L8R	−7.4
3AIC	−9.6	6CAM	−8.6	3OPU	−7.2
6TZL	−9.5	4TSH	−8.5	5ZA3	−6.8
3AIE	−9.5	3OIX	−8.4	3K8U	−6.6
2RI0	−9	5UQZ	−8.1	3PN8	−6.5
3BJV	−8.8	3EXT	−7.6	3CZC	−5.6

PDB, Protein Data Bank.

### Inhibition of Planktonic Growth

**Figure 2:**
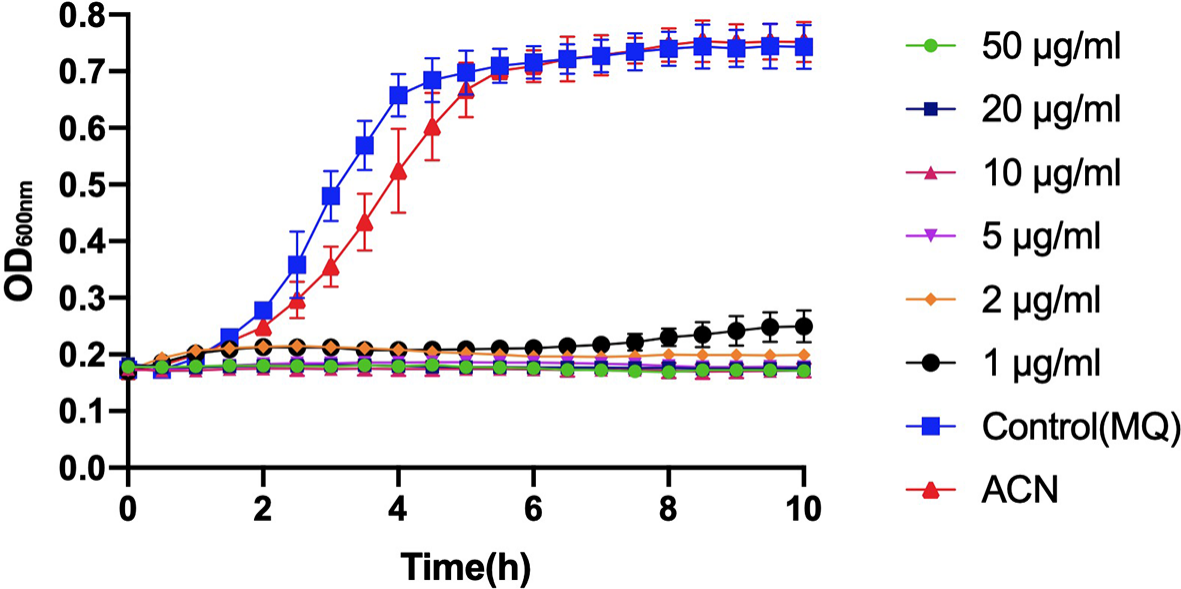
Growth curve of planktonic *Streptococcus mutans* UA159 incubated with 1, 2, 5, 10, 20, and 50 µg/ml of 1,3-bis[3,5-bis(trifluoromethyl)phenyl]urea (BPU). The same volumes of deionized water (MQ) and acetonitrile (ACN) were added to control groups. Values indicate means ± SDs from three independent experiments.

Compared to the control groups (treated with deionized water or acetonitrile), incubation with different levels of BPU significantly inhibited the growth of planktonic *S. mutans* UA159 in BHI broth (**Figure 2**). With a trace amount of drug in the culture (1 µg/ml), hardly any growth could be noticed in the bacterial culture.

### Inhibition of Biofilm Formation and Acid Production

**Figure 3:**
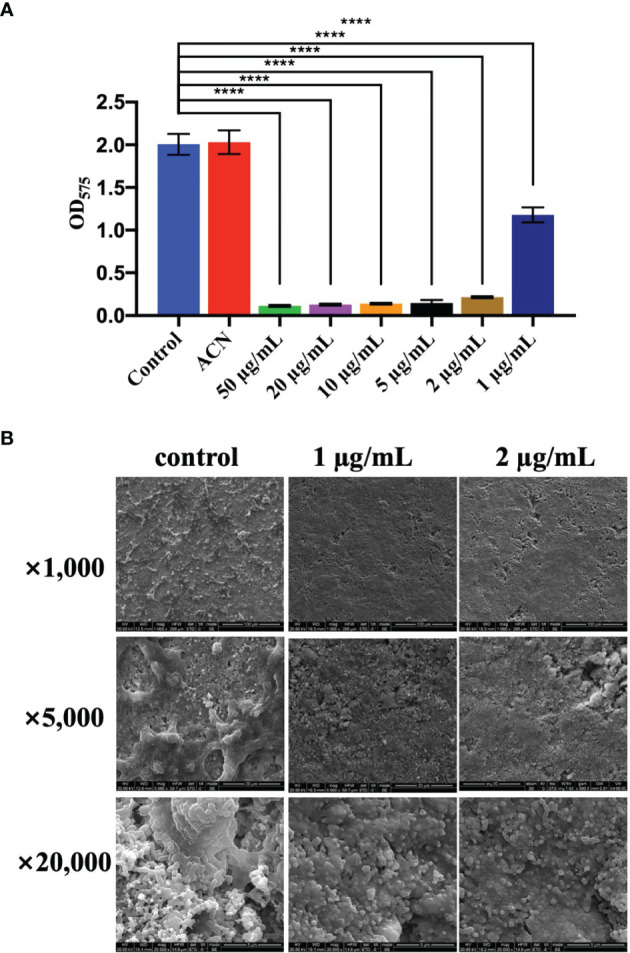
Effect of different levels of 1,3-bis[3,5-bis(trifluoromethyl)phenyl]urea (BPU) on biofilm formation of *Streptococcus mutans* UA159. **(A)** Biomass of biofilms quantified with crystal violet staining assay. **(B)** Representative SEM images of biofilms treated with 1 and 2 µg/ml of BPU or acetonitrile (control). Means ± SDs from three independent experiments are shown. **** indicates *p* < 0.0001.

Biomass of *S. mutans* UA159 biofilms treated with different levels of BPU was determined with crystal violet assay. As shown in **Figure 3A**, no significant difference was noticed between groups treated with deionized water and acetonitrile. Treatment of 1 µg/ml of BPU resulted in the loss of half of the biomass as compared to the control groups. Higher concentrations of BPU almost completely inhibited biofilm formation. Similar results were visualized with SEM examination (**Figure 3B**). Biofilms on sHA discs treated with 1 and 2 µg/ml of BPU were much thinner as compared to the control group. While biofilms of the control group appeared dense and highly stereoscopic, biofilms treated with BPU seemed plainer with more porous structures and less extracellular matrix in the system (**Figure 3B**).

**Figure 4:**
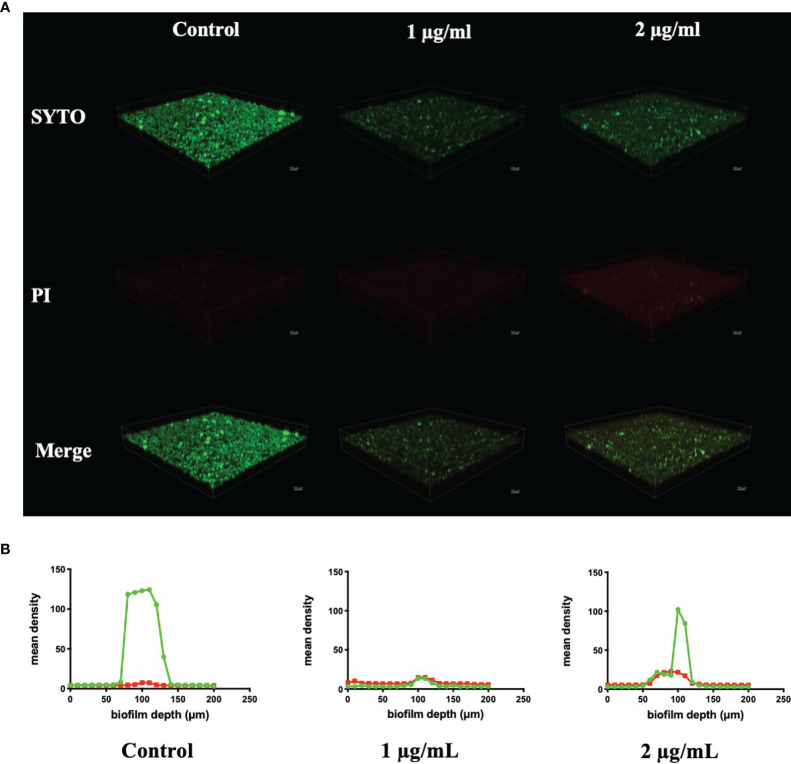
Live/dead staining of *Streptococcus mutans* UA159 biofilms treated with 1 and 2 µg/ml of 1,3-bis[3,5-bis(trifluoromethyl)phenyl]urea (BPU). **(A)** Representative three-dimensional visualization of live (green) and dead (red) cells in *S. mutans* biofilms formed on saliva-coated hydroxyapatite (sHA) discs. **(B)** Mean density of green and red fluorescence throughout *S. mutans* biofilms quantified using ImageJ software.

CLSM also found severe inhibition of biofilm formation and maturation by BPU (**Figure 4**). Strong green fluorescence in the control group (treated with acetonitrile) indicated a large number of viable cells in the biofilms. Biofilms treated with BPU exhibited significantly less green fluorescence and enhanced red fluorescence as compared to the control group (**Figure 4A**). Quantified mean fluorescence intensity confirmed that the signal of green fluorescence was remarkably lower in BPU-treated groups when compared with the control group (**Figure 4B**).

**Figure 5:**
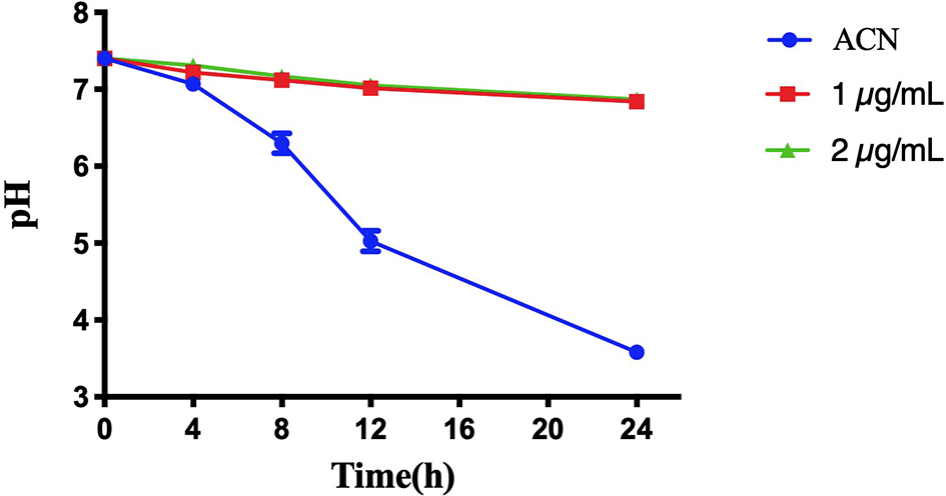
pH change of the supernatant of *Streptococcus mutans* UA159 biofilms treated with or without 1,3-bis[3,5-bis(trifluoromethyl)phenyl]urea (BPU). Values are presented as means ± SDs from three independent experiments. ACN, acetonitrile.

**Figure 5** shows the pH change of the supernatant of *S. mutans* biofilms treated with or without BPU within 24 h. As expected, biofilms treated with acetonitrile (control) were able to decrease environmental pH from 7.40 to 3.58 ± 0.03 within 24 h. Once treated with BPU, very little pH drop was noticed. At 24 h, pH of supernatant was similar for the 1 µg/ml and 2 µg/ml groups (pH 6.84 ± 0.08 and 6.87 ± 0.02, respectively).

### Inhibition of Exopolysaccharide Synthesis by *Streptococcus mutans* Biofilms

**Figure 6:**
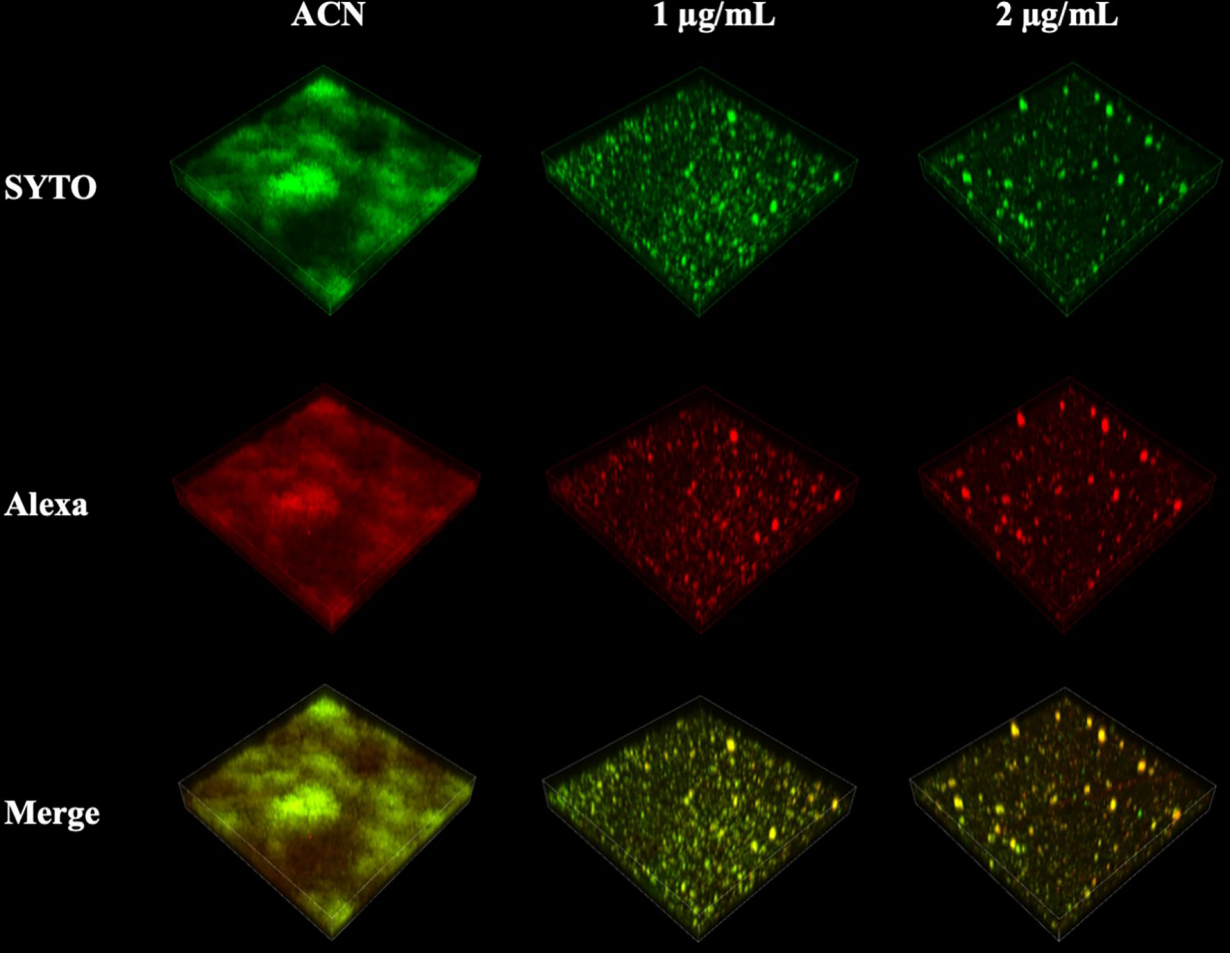
Confocal laser scanning microscopy (CLSM) images of *Streptococcus mutans* biofilm exopolysaccharide (EPS) synthesis affected by 1,3-bis[3,5-bis(trifluoromethyl) phenyl]urea (BPU). Green, bacterial cells (stained with SYTO 9); red, EPS (stained with Alexa 647). Representative three-dimensional images are exhibited.

EPS synthesis by *S. mutans* biofilms was examined using CLSM analysis and the anthrone–sulfuric method. CLSM images clearly showed that treatment of 1 and 2 µg/ml of BPU disrupted the ability of biofilms to synthesize EPS. Along with the decrease in EPS, the number of bacterial cells in biofilms also reduced significantly (**Figure 6**). While the control group exhibited thick, dense bacterial aggregations, groups treated with BPU appeared to be more dispersed with much thinner structures and fewer extracellular matrix connections (**Figure 6**).

The amount of EPS synthesized by biofilms treated with or without BPU was further examined using the anthrone–sulfuric method. Groups treated with deionized water and acetonitrile synthesized 0.53 ± 0.06 and 0.51 ± 0.06 mg/ml of EPS, respectively. The amount of EPS from two experimental groups (treated with 1 and 2 µg/ml of BPU) was both below the detection limit.

### Transcriptomic Analysis of *Streptococcus mutans* Treated With Diaryl Urea Derivative

**Figure 7:**
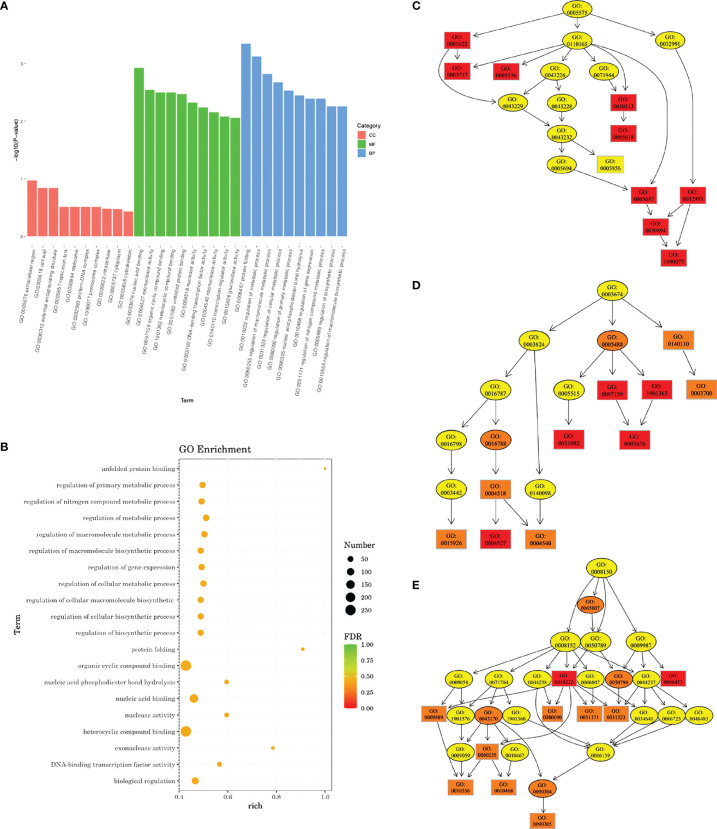
Gene Ontology (GO) enrichment analysis of transcriptomic profile. **(A)** Bar plot showing the most significantly enriched GO terms. CC, cellular component; MF, molecular function; BP, biological process. **(B)** Rich factors of 20 GO terms with the smallest false discovery rate (FDR) values. Directed acyclic graph (DAG) in cellular component **(C)**, molecular function **(D)**, and biological process **(E)**. Deeper color indicates a higher level of enrichment. GO accession number is shown.

Transcriptomic analysis revealed that a total of 701 DEGs were identified in the experimental group, including 270 upregulated genes and 431 downregulated genes. GO enrichment analysis indicated that a total of 68 GO terms involved in molecular function and biological processes were affected. A rich factor was calculated for 20 GO terms with the smallest false discovery rate (FDR) values (**Figure 7**). The results showed that GO term unfolded protein binding, protein folding, and exonuclease activity had the largest rich factors. A directed acyclic graph (DAG) was used to further display the relationship of the enriched GO terms. In cellular component, while not statistically significant, most differences were found in the cytoplasm (GO: 0005737), extracellular region (GO: 0005578), cell wall (GO: 0005618), and primosome complex (GO: 1990077). In molecular function, the difference was mostly concentrated in exonuclease activity (GO: 0004527), unfolded protein binding (GO: 0051082), and nucleic acid binding (GO: 0003676). In biological process, genes associated with protein folding (GO: 0006437), regulation of primary metabolic process (GO: 0080090), regulation of nitrogen compound metabolic process (GO: 0051171), and cellular nitrogen compound metabolic process (0031323) were most significantly differentially expressed (**Figure 7**).

We further looked into DEGs related to the regulation of metabolic processes and DEGs related to the regulation of macromolecule metabolic processes (**Supplementary Figures 1, 2**; **Supplementary Tables 1, 2**). Eleven genes involved in the regulation of metabolic process were upregulated, and 59 genes were downregulated. Most of them were associated with transcriptional regulation, sucrose metabolism, and nitrogen metabolism. For regulation of macromolecule metabolic process, 10 genes were upregulated and 58 downregulated. Among them were genes involved in EPS synthesis (*sacR*, *scrR*, and *msmR*), stress tolerance (*rex*, *perR*, *spxA*, *clpE*, and *ciaR*), and nitrogen metabolism (*glnB*, *glnR*, *ciaR*, and *clpE*).

**Figure 8:**
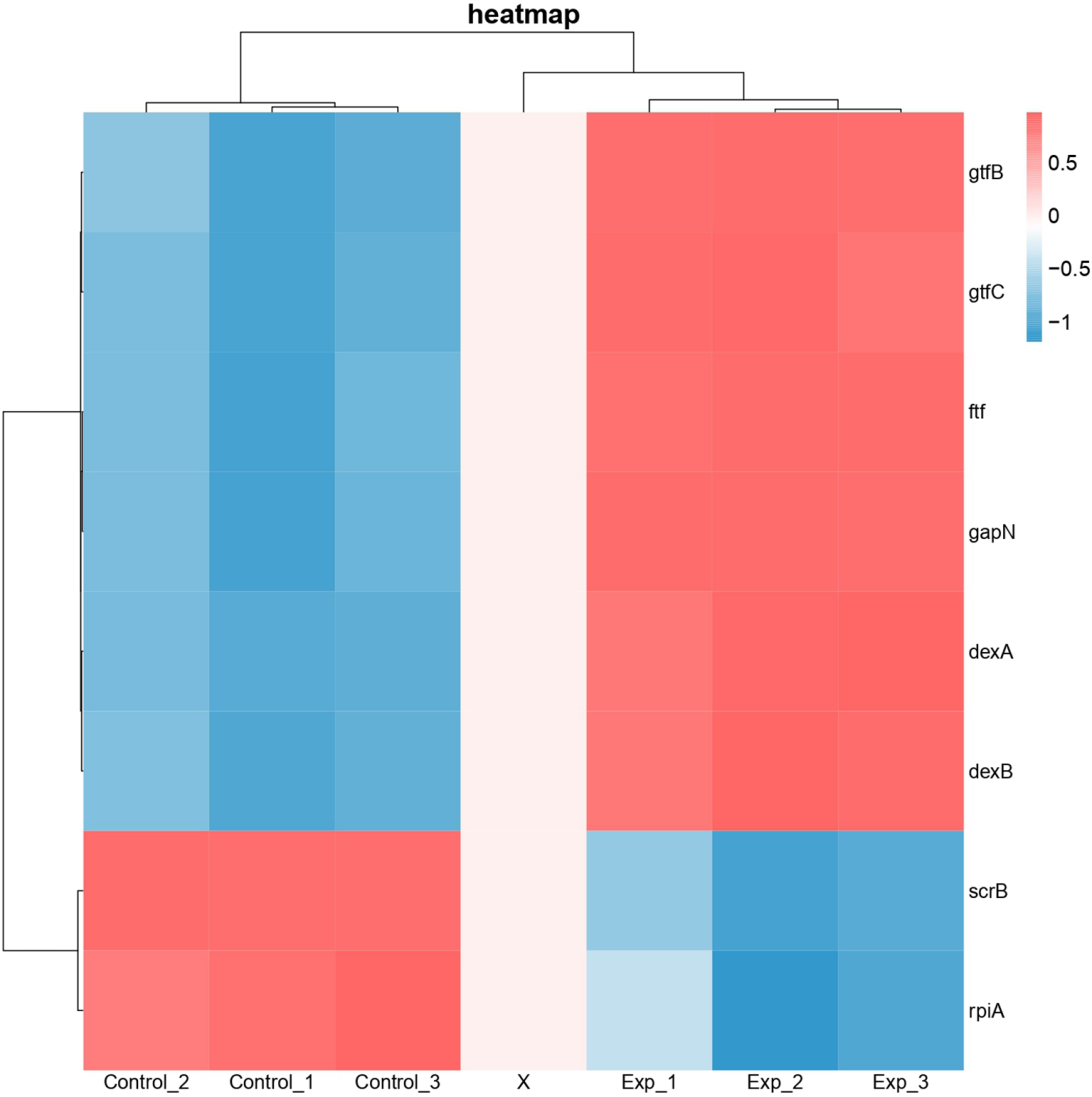
Heatmap of differentially expressed genes associated with protein functions of 3AIC, 2ZID, and 3M7V. Control groups were treated with acetonitrile. Experimental groups were treated with 2 µg/ml of 1,3-bis[3,5-bis(trifluoromethyl)phenyl]urea (BPU). Genes *gtfB*, *gtfC*, *ftf*, *dexA*, *dexB*, and *scrB* are involved in exopolysaccharide biosynthesis. Genes *gapN* and *rplA* are involved in pentose phosphate pathway. Blue indicates lower expression, and red indicates higher expression.

Differential expression of genes associated with protein functions of 3AIC, 2ZID, and 3M7V was also examined (**Figure 8**). As 3AIC and 2ZID are both proteins involved in EPS synthesis, genes associated with EPS synthesis (*gtfB*, *gtfC*, *ftf*, *dexA*, *dexB*, and *scrB*) were selected. For 3M7V, its encoding gene (*deoB*) and two related genes involved in the pentose phosphate pathway (*gapN* and *rpiA*) were selected. The heatmap shows that most genes involved in EPS synthesis, including encoding genes of 3AIC and 2ZID (*gtfC* and *dexB*), were upregulated. The encoding gene of 3M7V (*deoB*) was not found differentially expressed. Its related genes showed different regulatory directions (*gapN* was upregulated and *rpiA* was downregulated).

## Discussion

Small molecule compounds have been extensively explored as potent anti-caries agents targeting caries-associated bacteria. In this study, we described the antimicrobial and antibiofilm effects of a novel diaryl urea derivative, BPU. *In silico* simulation indicated that the small molecule compound had the potential to bind the key enzymes involved in EPS biosynthesis and the pentose phosphate pathway. The transcriptomic study suggested that except for EPS synthesis, nitrogen metabolism and stress-responsive pathway were also affected by the small molecule.

Diaryl rings have previously been suggested to be able to bind specific bacterial proteins (Limban et al., 2020; Sroor et al., 2021) and act as enzyme inhibitors. Our *in silico* molecular docking also found strong binding potential between BPU and several enzymes involved in the biological processes of *S. mutans*. Interestingly, 2 of the top 3 hits (2ZID and 3AIC) are involved in the biosynthesis of EPS. EPS is the main component of the extracellular matrix of bacterial biofilms, and the ability to synthesize EPS is a vital virulence factor of *S. mutans* (Koo et al., 2013; Krzysciak et al., 2014; Klein et al., 2015). Protein 2ZID is a dextran glucosidase hydrolyzing the α-1,6-glucosidic linkage of α-1,6-d-glucans and derived oligosaccharides (Hondoh et al., 2008). Protein 3AIC is a glucosyltransferase responsible for water-insoluble and water-soluble glucan syntheses in *S. mutans* (Ito et al., 2011). The binding mode prediction shows that the diaryl urea derivative molecule binds to either substrate binding pocket or catalytic domains of the two proteins. This indicates that the binding of the molecule can competitively inhibit the activity of 3AIC and 2ZID. A review of the literature shows that a number of 3AIC inhibitors have been proved effective in inhibiting biofilm formation of *S. mutans* and thus caries development (Ren et al., 2016; Zhang et al., 2017; Lin et al., 2021). In this study, we noticed significantly less biofilm formation after BPU treatment with crystal violet assay and SEM observation. CLSM examination and EPS quantification further confirmed that production of EPS was severely impaired by the treatment of BPU. Further transcriptomic analysis revealed that both genes encoding 3AIC and 2ZID (*gtfC* and *dexB*) were upregulated in *S. mutans* treated with BPU. The expression of several genes with associated functions, including *gtfB*, *ftf*, and *dexA* (Li and Burne, 2001; Koo et al., 2013; Yang et al., 2019), was also upregulated. This could be a result of negative feedback of the inhibited activity of corresponding proteins. Accordingly, genes encoding related transcription repressors (*sacR* and *scrR*) were downregulated (**Supplementary Table 1**) (Siegers and Entian, 1995; Hiratsuka et al., 1998), further confirming that bacterial cells were combating the inhibitive effect of BPU on EPS synthesis.

Our results suggest that the treatment of BPU not only inhibits biofilm formation but also affects planktonic growth. An examination of the transcriptomic profile revealed that, except for the EPS synthesis pathway, multiple genes involved in stress response were differentially expressed. Previous studies have found that the function of stress response proteins is important for bacterial physiology. Deficiency of oxidative stress response regulator Rex has been found to lead to an extended lag phase of planktonic growth of *S. mutans* (Bitoun et al., 2011). Moreover, expression of *gtfC* was also found significantly differentially expressed due to deficiency of Rex, leading to decreased biofilm formation and altered biofilm morphology (Bitoun et al., 2011). In our study, the downregulation of *rex* could be associated with decreased planktonic growth and biofilm formation. The regulatory effect of SpxA has been implicated in oxidative stress response, antibiotic response, and biofilm formation (Pamp et al., 2006; Kajfasz et al., 2015; Nilsson et al., 2019). Similarly, two genes involved in oxidative and thermal stress responses, *clpE* and *ciaR*, have the ability to affect the biofilm formation of *S. mutans* (Zhu et al., 2017; Biswas et al., 2021). All three abovementioned genes involved in stress response were found to be downregulated in the current study after BPU treatment (**Supplementary Table 2**). The involvement of stress response indicates new targets for novel anti-caries agents.

Additionally, relatively abundant changes in nitrogen metabolism in the BPU-treated group were observed in the transcriptomic profile. By metabolizing nitrogen-containing compounds, mainly amino sugars such as glucosamine and *N*-acetylglucosamine, bacteria are provided with essential materials for macromolecule synthesis (Ardin et al., 2014). The ability to utilize amino sugars is also closely associated with the competitiveness of bacteria to thrive in the oral cavity (Chen et al., 2020). The current study reports the downregulation of genes encoding a membrane ammonium transporter (*glnB*) (Ardin et al., 2014) and a glutamine synthetase regulator (*glnR*) (Chen et al., 2010). Results of *in silico* molecular docking suggest that a key factor affecting cellular entrance of amino acid, NagB (2RI0), has a relatively high binding potential with BPU molecule (−9 kcal/mol, **Table 2**) (Kawada-Matsuo et al., 2012). The potential inhibition of NagB together with the downregulation of associated genes supports that nitrogen metabolism may be inhibited by the diaryl urea derivative. The resulting decreased synthesis of virulence-related factors, including cell surface protein antigen and glucosyltransferase, can lead to less bacterial aggregation and thus less biofilm formation (Kawada-Matsuo et al., 2012).

The molecular docking analysis suggests that the BPU molecule has the potential to bind to an enzyme involved in the pentose phosphate pathway (3M7V, DeoB). According to previous studies, DeoB acts as a vital role in the pentose phosphate pathway, providing a precursor for phosphoribosyl pyrophosphate, which is involved in histidine and purine biosynthesis (Tozzi et al., 2006; Xue et al., 2012). However, we did not notice significant changes in expression of the encoding gene *deoB* in groups treated with BPU in the transcriptional profile. Nor did we find an obvious change in the pentose phosphate pathway according to GO enrichment analysis. One explanation is that while a strong binding potential exists between the molecule and the protein, the binding site may not be close to the essential substrate or metal ion binding site. Therefore, the activity of 3M7V and the pentose phosphate pathway may not be significantly affected. Till now, very little information on the functional groups of 3M7V is available, and the effect of the diaryl urea derivative on the protein and its associated pathway requires further validation.

Based on the structure of BPU, previous literature predicted that it may affect cell wall biosynthesis and membrane permeability (Nelson et al., 2015). We were expecting to find changes in GO terms involved in cellular component using GO enrichment analysis. However, no significant alteration was found. We looked specifically into genes involved in the GO term “cell wall” and found 3 DEGs (**Supplementary Table 3**). What may be of interest is that 2 upregulated genes, *spaP* and *dexA*, encode cell surface proteins, which are important for the adherence of *S. mutans* to the tooth surface (SpaP) and dextran-dependent aggregation (DexA) (Goldschmidt et al., 1990; Yang et al., 2019). This is again in accordance with the upregulation of other EPS synthesis-related genes. The results suggest that multiple steps and factors involved in or associated with EPS synthesis are influenced by the diaryl urea derivative.

In the present study, we noticed a strong inhibitive effect of BPU on growth, biofilm formation, and acidogenesis of *S. mutans*. Instead of being dosage-dependent, the inhibition seemed to be similar among groups treated with different concentrations of BPU. In other words, the inhibitory effect seemed to be an “ON/OFF” mode. In fact, this is not the first time that we found this kind of inhibition mode in *S. mutans*. One possible explanation is that the small molecule affects essential factor(s) involved in bacterial growth and/or metabolism. For example, our previous study confirmed that different concentrations of fluoride led to similar levels of inhibition of enolase activity, a key enzyme in glycolysis, in an *S. mutans* strain (Liao et al., 2018). As we found alterations in several biological signs of progress including sugar metabolism, nitrogen metabolism, and stress response, it is difficult to identify the major switch for the “ON/OFF” inhibition. Further knockout studies could help disclose the inhibitory “switch” of BPU.

In conclusion, our current study has confirmed the strong antimicrobial and antibiofilm effects of a novel urea derivative molecule, BPU, against caries-associated *S. mutans*. Structure-based *in silico* prediction together with the transcriptomic study has suggested the involvement of several biological processes affected by the small molecule. It is predicted that the treatment of BPU could alter the expression of genes involved in EPS synthesis, stress response, and nitrogen metabolism, leading to immediate and strong inhibition of bacterial growth, acid production, and biofilm formation. Our work illustrates that BPU is a promising agent to be used in caries prevention.

## Data Availability Statement

The transcriptomic data presented in the study are deposited in NCBI SRA database with accession number PRJNA819867.

## Author Contributions

YL and FY contributed to the conception and design of the study. YL and MZ conducted the experiments. YL performed the transcriptomic analysis. All authors contributed to the interpretation of the data. YL wrote the first draft of the manuscript. All authors contributed to the manuscript revision and approved the submitted version.

## Funding

This work was supported by the National Natural Science Funds (grant number 82001035), the Jiangsu Natural Science Funds (grant number SBK2020041847), the Nanjing Clinical Research Center for Oral Diseases (grant number 2019060009), and the "3456" Cultivation Program for Junior Talents of Nanjing Stomatological School, Medical School of Nanjing University (grant number).

## Conflict of Interest

The authors declare that the research was conducted in the absence of any commercial or financial relationships that could be construed as a potential conflict of interest.

## Publisher’s Note

All claims expressed in this article are solely those of the authors and do not necessarily represent those of their affiliated organizations, or those of the publisher, the editors and the reviewers. Any product that may be evaluated in this article, or claim that may be made by its manufacturer, is not guaranteed or endorsed by the publisher.
