# Supplementary figures and images for: Diaryl Urea Derivative Molecule Inhibits Cariogenic Streptococcus mutans by Affecting Exopolysaccharide Synthesis, Stress Response, and Nitrogen Metabolism
Source: Front Cell Infect Microbiol. 2022 May 10;12:904488. doi: 10.3389/fcimb.2022.904488 (PMC9127343; doi:10.3389/fcimb.2022.904488)

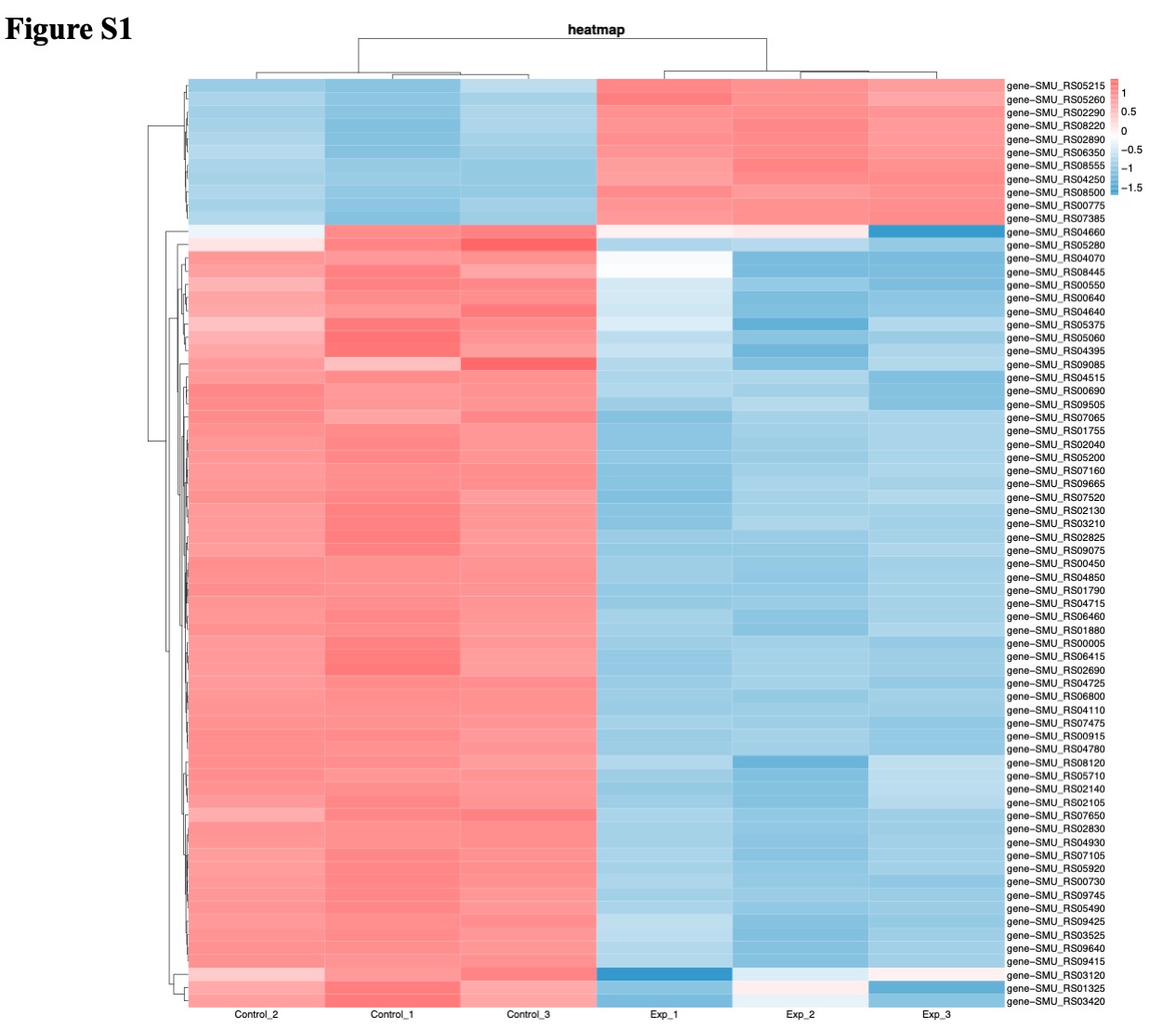

Supplement: Supplementary file 1 [file Image_1.jpeg]

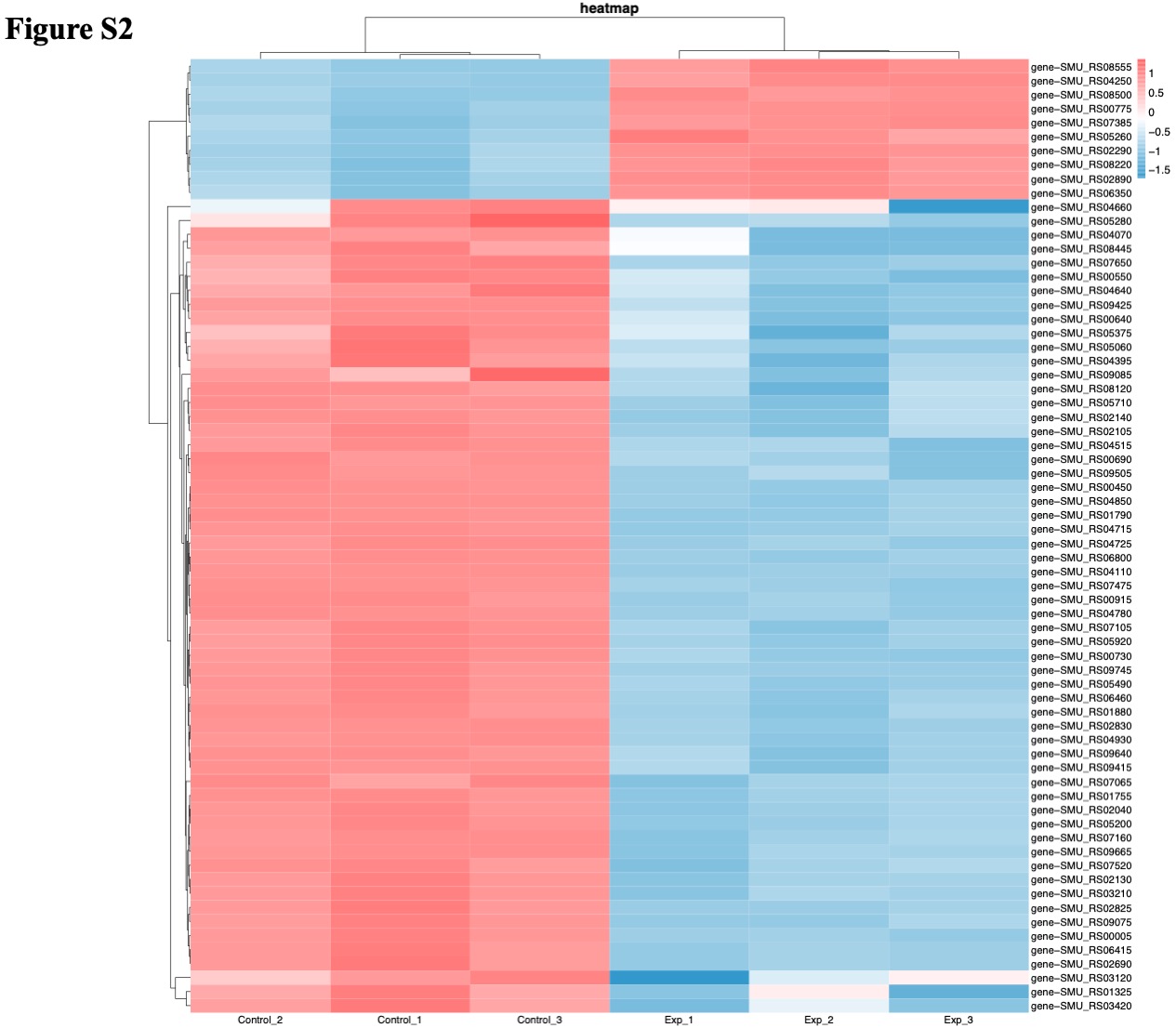

Supplement: Supplementary file 2 [file Image_2.jpeg]
